# Comparative Study of Endoscopic Treatment for Intrahepatic and Common Bile Duct Stones Using Peroral Cholangioscopy

**DOI:** 10.3390/jcm13185422

**Published:** 2024-09-12

**Authors:** Yuri Sakamoto, Yohei Takeda, Taro Yamashita, Yuta Seki, Shiho Kawahara, Takayuki Hirai, Noriyuki Suto, Takuya Shimosaka, Wataru Hamamoto, Hiroki Koda, Takumi Onoyama, Kazuya Matsumoto, Kazuo Yashima, Hajime Isomoto, Naoyuki Yamaguchi

**Affiliations:** 1 Division of Gastroenterology and Nephrology, Department of Multidisciplinary Internal Medicine, Faculty of Medicine, Tottori University, Tottori 683-8504, Japan; yuri.sakamoto@tottori-u.ac.jp (Y.S.);; 2 Department of Endoscopy, Nagasaki University Hospital, Nagasaki 852-8501, Japan

**Keywords:** bile ducts, bile stones, cholangitis, common bile duct, endoscopic retrograde cholangiopancreatography, intrahepatic bile duct, peroral cholangioscopy

## Abstract

**Objectives**: Although peroral cholangioscopy has improved the endoscopic treatment of difficult stones, the treatment of intrahepatic stones remains challenging. The incidence of cholangitis is high when peroral cholangioscopy is used to treat intrahepatic stones. This study aimed to investigate the efficacy and safety of endoscopic treatment with peroral cholangioscopy for intrahepatic and common bile duct stones. **Methods**: Patients aged ≥20 years, who underwent endoscopic treatment with peroral cholangioscopy for intrahepatic or common bile duct stones at Tottori University Hospital from January 2016 to December 2022, were retrospectively evaluated to determine the efficacy and safety of the treatment. **Results**: Overall, 70 patients were included in this study: 22 in the intrahepatic stone group and 48 in the common bile duct stone group. Stones were smaller (8 vs. 17.5 mm, *p* < 0.001) and more numerous (*p* = 0.016) in the intrahepatic stone group than in the common bile duct stone group. Although the common bile duct stone group exhibited a higher rate of complete stone clearance in the first session, no significant differences were observed in the final results. The intrahepatic stone group had a higher incidence of cholangitis (36% vs. 8%, *p* = 0.007); however, all cases were mild. **Conclusions**: Endoscopic treatment with peroral cholangioscopy for intrahepatic stones may be associated with a higher incidence of cholangitis than that for common bile duct stones. Since saline irrigation may contribute to the development of cholangitis, it is important to be aware of intraductal bile duct pressure when performing peroral cholangioscopy.

## 1. Introduction

The definition of “difficult stones” varies across reports as the condition is described based on the devices and techniques available at the time and in each place [1]. According to Yasuda et al., the anatomical situation (altered anatomy, periampullary diverticulum), the character (size, number), and location (intrahepatic stones, above strictures, or impacted in the bile duct or cystic duct) of the stones, as well as the patient’s condition (very poor general condition, old age, or bleeding tendency), contribute to the definition of difficult stones [1].

Intrahepatic stones are difficult stones. While intrahepatic stones were traditionally considered prevalent in Southeast Asia, their incidence has increased in Western countries owing to increased immigration [2]. Intrahepatic stones pose risks, such as sepsis, liver abscesses, and bile duct cancer [3,4]. The conventional treatment of intrahepatic stones often involves liver resection or percutaneous transhepatic therapy [5,6]. In recent years, with the advent of peroral cholangioscopy (POCS) and electrohydraulic lithotripsy (EHL), bile duct stones, which were previously difficult to treat, can now be treated endoscopically in an increasing number of cases. EHL or laser lithotripsy under POCS is particularly effective for managing large stones in the common bile duct; however, the treatment of intrahepatic stones remains challenging [7]. Additionally, POCS-assisted EHL for intrahepatic stones is associated with a higher incidence of cholangitis. Therefore, it is necessary to exercise caution and meticulously perform the procedure in experienced medical facilities [8].

Reports on the endoscopic treatment of intrahepatic stones are scarce. Previous studies have examined the endoscopic treatment of difficult stones, including a few cases of intrahepatic stones; however, no studies have focused specifically on the results and efficacy of intrahepatic stone treatment [9]. Factors that increase the difficulty of stone removal by conventional endoscopic retrograde cholangiopancreatography (ERCP) reportedly include Mirizzi syndrome, intrahepatic stones, a stricture that is distal to a stone, and a narrow bile duct–stone ratio [9]. Intrahepatic stones typically satisfy all these conditions other than Mirizzi syndrome.

**Figure 1:**
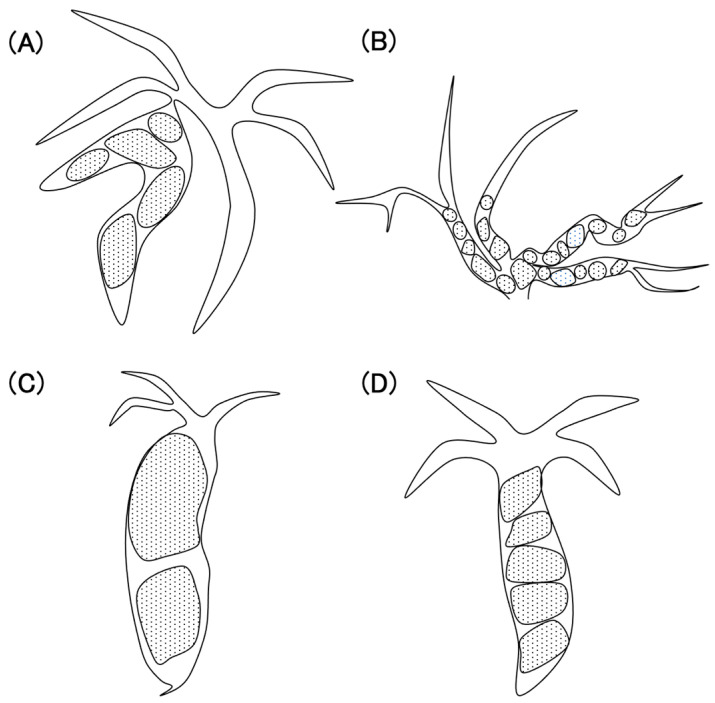
(**A**) Multiple stones caused by bile duct stenosis. (**B**) Stones obstructing the intrahepatic bile duct. (**C**) Large stones in the common bile duct. (**D**) Multiple stones in the common bile duct.

We consider the characteristics of intrahepatic stones and common bile duct stones to be very different; thus, they should be studied separately when examining the efficacy and safety of endoscopic treatment. Typical cases of difficult-to-treat intrahepatic and common bile duct stones at our hospital are presented herein. Intrahepatic stones are often seen in patients with bile duct narrowing due to conditions such as primary sclerosing cholangitis or postoperative scars (Figure 1A) or, in cases warranting EHL, due to the obstruction of the intrahepatic bile duct, attributed to anastomotic stricture (Figure 1B). However, difficult-to-treat common bile duct stones are often large stones that cannot be grasped with a basket catheter (Figure 1C) or stacked stones, making it challenging to employ stone removal devices (Figure 1D). Although reports of the endoscopic treatment of difficult stones are widespread, few studies have compared the results of the endoscopic treatment of intrahepatic stones and common bile duct stones. We focused on the differences between intrahepatic stones and common bile duct stones and examined and compared the treatment outcomes of POCS-assisted EHL to evaluate its efficacy and safety in treating both types of stones.

## 2. Materials and Methods

### 2.1. Study Design and Participants

This was a single-center, retrospective, and observational study. Participants were enrolled between January 2016 and December 2022 at the Tottori University Faculty of Medicine Hospital. This study was approved by the Tottori University Faculty of Medicine Hospital (approval no. 1508A024). Patients aged ≥20 years, who were treated endoscopically for intrahepatic or common bile duct stones using POCS, were eligible for inclusion, and patients who did not consent to the study were excluded. Consent for inclusion in this study was obtained on an opt-out basis.

### 2.2. Materials

The TJF-Q290V or JF-260V and CF-HQ290ZI endoscopes for surgically altered anatomy (Olympus Medical Science Corporation, Tokyo, Japan) were used in this study. The cholangioscope was a SpyGlass^TM^ DS II (hereafter referred to as “SpyGlass”) (Boston Scientific Corporation, Marlborough, MA, USA, and EHL was conducted using the Autolith^®^ Touch system (Boston Scientific Corporation, Marlborough, MA, USA). In cases with no contraindications, diclofenac suppositories were administered to prevent post-ERCP pancreatitis. After February 2019, prophylactic antibiotics were administered twice, immediately before and 5 h after the procedure (until January 2019, the prophylactic administration of antibiotics was left to the discretion of the attending physician).

### 2.3. Procedure

**Figure 2:**
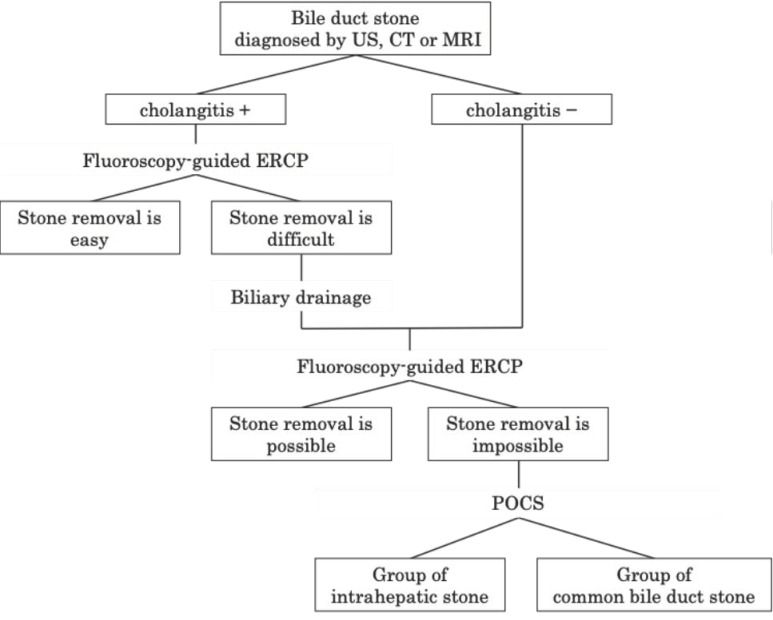
Procedure for treatment selection. Abbreviations: US; ultrasound, CT; computed tomography, MRI; magnetic resonance imaging, ERCP; endoscopic retrograde cholangiopancreatography, POCS; peroral cholangioscopy.

This study focused on cases in which POCS was performed after fluoroscopy-guided ERCP failed to treat bile duct stones or in which imaging findings indicated that fluoroscopy-guided treatment was likely to fail. Initially, biliary drainage was performed using conventional ERCP for patients presenting with cholangitis at their initial visit to our hospital. POCS was performed in suitable cases after cholangitis was alleviated (Figure 2). The number of procedures was counted from the first POCS session until complete stone removal or the termination of treatment.

Fluoroscopy-guided treatment involves papillary procedures (e.g., endoscopic sphincterotomy, endoscopic papillary balloon dilation, or endoscopic papillary large-balloon dilation) and stone extraction using a basket or balloon catheter. In POCS-assisted treatment, after the papillary procedure, the SpyGlass was inserted into the bile duct for direct visualization to confirm the presence of stones. These were crushed using EHL if necessary.

The group treated for stones that formed in the left and right hepatic ducts and upstream of these was defined as the intrahepatic stone group (IH group), and the group treated for stones formed in the common bile duct was defined as the common bile duct stone group (CBD group).

### 2.4. Evaluation of Outcomes

We retrospectively evaluated the procedure time, adverse events in the first session, and the complete stone removal rate. Procedure time was measured from scope insertion until removal. The adverse events were graded according to the American Society for Gastrointestinal Endoscopy Lexicon Severity Grading System [10]. Complete stone removal was defined as the absence of stones upon cholangiography at the end of endoscopic treatment. The size and number of the stones were measured and counted using cholangiography or computed tomography.

### 2.5. Statistical Analysis

Descriptive statistics are presented as medians and ranges. Welch’s *t*-test was used to compare the medians of continuous variables, and Fisher’s exact test was used to compare the proportions of categorical variables in the groups. Statistical significance was set at *p* < 0.05. All statistical analyses were performed using the EZR software (version 4.2.1) (Saitama Medical Center, Jichi Medical University, Saitama, Japan) [11].

## 3. Results

### 3.1. Patient Background

Table 1 presents the patients’ characteristics. This study included 70 patients, including 22 with intrahepatic stones and 48 with common bile duct stones. There were 40 males and 30 females, with a median age of 79 (26–97) years, and 6 patients with surgically altered anatomy were enrolled. Surgically modified anatomy included four cases of modified child, one case of Roux-en-Y anastomosis following total gastrectomy, and one case of choledocho-jejunostomy. In this study, Billroth I reconstruction was not included in the surgically modified anatomy. The median stone size was 14 (3–45) mm, with 17 patients (24%) having 1 stone, 22 (31%) having 2–5 stones, and 31 (44%) having ≥6 stones. Difficulties in treatment were attributed to multiple stones in 24 cases (34%), large stones in 19 cases (27%), stenosis in 11 cases (16%), the requirement of endoscopic papillary balloon dilation as a papillary procedure in 4 cases, surgically altered anatomy in 2 cases (3%), and other reasons in 10 cases (14%). Table 1 also shows a comparison of the characteristics of the IH and CBD groups. Participants in the IH group were younger (69 vs. 83 years of age, *p* = 0.006). There was no difference in the sex ratio. Surgically altered anatomy was more common in the IH group (23% vs. 2%, *p* = 0.03). The IH group included four cases of modified child and one case of Roux-en-Y anastomosis, and the CBD group included one case of choledocho-jejunostomy. In the IH group, the stones were smaller (8 mm vs. 17.5 mm, *p* < 0.001) and more numerous (*p* = 0.016) than those in the CBD group. The difficulty in stone removal was mainly attributable to the presence of multiple stones and stenosis in the IH group, and the presence of multiple and large stones in the CBD group. 

### 3.2. Outcomes

Table 2 presents the treatment outcomes. Patients with untreated papillae accounted for <20% in both groups, indicating that many had previously undergone endoscopic procedures; however, complete stone removal was difficult. There were no differences in the papillary interventions performed in this study. The treatment times were similar in both groups. The complete stone removal rate tended to be higher in the CBD group than in the IH group in the first session (59% vs. 79%, *p* = 0.06); however, after multiple treatments, both groups performed well (95% vs. 100%, *p* = 0.3). The number of treatment sessions tended to be higher in the IH group.

### 3.3. Complications

Overall, the complications included 12 cases of cholangitis and 3 cases of pancreatitis. No cases of bleeding or perforation were observed. The incidence of cholangitis was significantly higher in the IH group (8/22 [36%] vs. 4/48 [8%], *p* = 0.007). No difference was observed in the incidence of pancreatitis between the two groups (2/22 [9%] vs. 1/48 [2%], *p* = 0.24). All cholangitis and pancreatitis cases were mild and were resolved using conservative treatment.

The factors associated with the occurrence of cholangitis were examined in the IH group (Table 3). Stones in patients who developed cholangitis tended to be smaller and more numerous. Additionally, patients who had endoscopic sphincterotomy performed on untreated papillae experienced cholangitis. No difference was observed in the incidence of cholangitis before and after the initiation of prophylactic antibiotic administration in February 2019.

## 4. Discussion

### 4.1. Assessment of Patient Background

As expected, the IH group had smaller and more numerous stones than the CBD group; we consider the causes of stone formation in the IH group to include anastomotic stenosis of the surgically altered anatomy, which also contributed to the age difference between the groups.

### 4.2. Assessment of Outcomes

**Figure 3:**
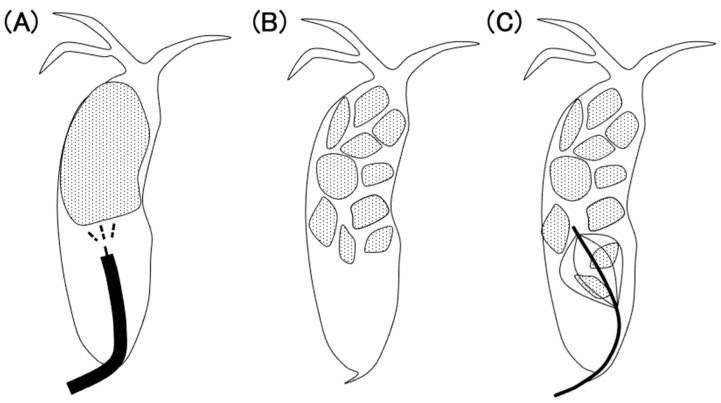
(**A**) Electrohydraulic lithotripsy (dashed lines) is performed under peroral cholangioscopy (black line). (**B**) Crushed stone. (**C**) Stone removal from the distal side by a basket catheter.

**Figure 4:**
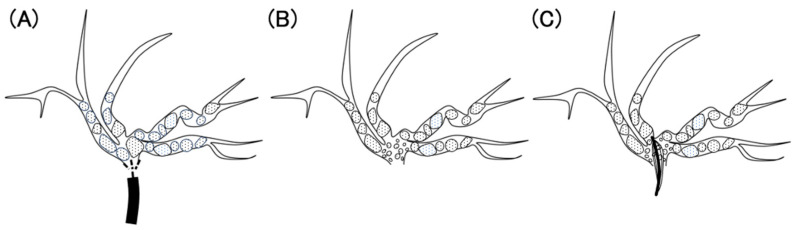
(**A**) Electrohydraulic lithotripsy (dashed lines) is performed under oral cholangioscopy (black line). (**B**) The bile duct is so narrow that lithotripsy can only be performed gradually. (**C**) A basket catheter cannot be expanded sufficiently.

Endoscopic treatment with POCS and EHL was effective in the IH and CBD groups. Compared to the CBD group, the IH group tended to require more procedures and the treatment of stones was more challenging in them. For cases with common bile duct stones, after the stones were partially crushed by EHL, fluoroscopic stone removal with a basket or balloon catheter was relatively easy (Figure 3). However, intrahepatic stones are often difficult to open using a basket or balloon catheter because the tapered bile duct is filled with stones (Figure 4). Therefore, it may not be possible to remove the stone sufficiently after crushing. If the stones remain, a plastic stent or endoscopic nasobiliary drainage tube should be placed to maintain the biliary outflow pathway in order to prevent cholangitis. In cases of bile duct stenosis, the placement of a plastic stent may improve the stenosis. After these treatments, adequately crushed stones have the potential to be passed naturally to some extent. With each successive procedure, the therapeutic process gradually becomes more manageable. Therefore, in cases with intrahepatic stones, it is more beneficial to prioritize safety over the aggressive pursuit of complete stone removal in a single session, opting instead for a steady and gradual approach, even if this takes time.

### 4.3. Assessment of Complications

**Figure 5:**
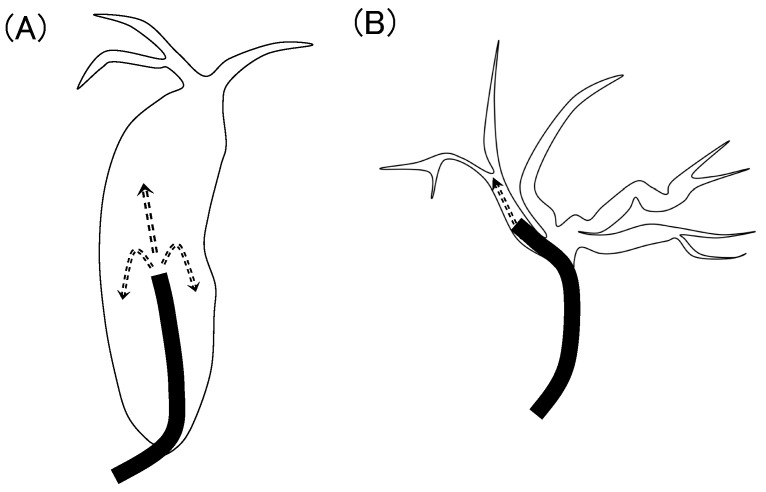
(**A**) The common bile duct; (**B**) the intrahepatic bile duct. (**A**) Saline perfusion pressure (arrows) is spread out owing to the large space. (**B**) The pressure is concentrated in one bile duct owing to the narrow space.

Notably, the incidence of cholangitis was higher in the IH group than in the CBD group. The incidence of cholangitis during POCS was 5.9–7.5% [12,13], whereas the incidence of cholangitis during regular ERCP was 0.57–1.4% [14,15]. The incidence of cholangitis reportedly increases with POCS. This increase was attributed to increased intraductal bile duct pressure due to saline irrigation [16]. Intrahepatic stones often occur in patients with bile duct stenosis and may be affected more by irrigation because the diameter of the intrahepatic bile duct is narrower than that of the common bile duct. As shown in Figure 5, the common bile duct is often dilated in the presence of large or multiple stones, and saline irrigation using SpyGlass distributes the pressure. If sufficient papillary treatment has already been performed, saline also flows out of the papillae; therefore, pressure in the common bile duct is less likely to increase. However, in cases with intrahepatic stones, the intrahepatic bile duct is narrow, and the pressure is concentrated in a specific branch. If the SpyGlass is wedged and a small amount of saline flows toward the papillary side, intrahepatic bile duct pressure is further increased.

In the IH group, patients who developed cholangitis had smaller and more numerous stones than those who did not develop cholangitis. Patients with small and numerous stones often have stones that fill their small bile ducts. When EHL is performed in such cases, sufficient quantities of water must be pumped to maintain the visual field. Sufficient aspiration before and after pumping is important to preventing overperfusion, which can increase intraductal bile duct pressure. The direct measurement of the biliary pressure during cholangioscopy is not yet possible. Therefore, when using SpyGlass in the intrahepatic bile duct, the operator must be careful not to raise the intraductal bile duct pressure excessively. The limitations of this study include its single-center retrospective design and possible variations in the endoscopists’ skill levels. Furthermore, the number of patients included in this study may have been insufficient, which could have introduced bias, especially in the subgroup analysis.

## 5. Conclusions

Endoscopic treatment with POCS is effective for intrahepatic and common bile duct stones. Intrahepatic stones may be highly susceptible to cholangitis during endoscopic treatment with POCS owing to their etiology and anatomy. Since saline irrigation may contribute to the development of cholangitis, it is important to pay attention to the intraductal bile duct pressure during POCS. Moreover, if the stones are sufficiently crushed by the EHL, they may pass naturally after undergoing endoscopic procedure; therefore, repeating the procedure may be more effective than aiming for complete stone removal in a single session.

## Figures and Tables

**Table 1:** Characteristics of the study’s participants.

Factors	All (*n* = 70)	IH (*n* = 22)	CBD (*n* = 48)	*p* Value
Clinical features				
Age, median	79 (26–97)	69 (26–90)	83 (38–97)	0.006
Sex, male/female	40/30	16/6	24/24	0.12
Surgically altered anatomy	6 (8.6%)	5 (23%)	1 (2%)	0.01
Stones				
Intrahepatic duct	22 (31%)			
Common bile duct	48 (69%)			
Largest stone size (mm), median	14 (3–45)	8 (3–21)	17.5 (5–45)	<0.001
Number of stones				
1	17 (24%)	2 (9%)	15 (31%)	0.016
2–5	22 (31%)	6 (27%)	16 (33%)	
>6	31 (44%)	14 (64%)	17 (35%)	
Reason for difficulty				
Multiple stones	24 (34%)	6 (27%)	18 (38%)	0.006
Large stone	19 (27%)	1 (5%)	18 (38%)	
Stenosis	11 (16%)	8 (36%)	3 (6%)	
EPBD	4 (6%)	0	4 (8%)	
Surgically altered anatomy	2 (3%)	2 (9%)	0	
Others	10 (14%)	5 (23%)	9 (19%)	

IH: intrahepatic stones, CBD: common bile duct stones, EPBD: endoscopic papillary balloon dilation.

**Table 2:** Treatment outcomes in the study’s groups.

Factors	IH (*n* = 22)	CBD (*n* = 48)	*p* Value
Untreated papillae	4 (18%)	9 (19%)	1
Papillary intervention			
EST	2 (9%)	11 (23%)	0.5
EPBD	5 (23%)	5 (10%)	
EPLBD	2 (9%)	9 (19%)	
None	13 (59%)	23 (48%)	
Procedure time (min), median	105.5	100	0.5
Complete stone removal			
First session	13 (59%)	38 (79%)	0.06
Final session	21 (95%)	48 (100%)	0.3
Number of sessions, median	1 (1–7)	1 (1–2)	0.08

IH: intrahepatic stone, CBD: common bile duct stones, EST: endoscopic sphincterotomy, EPBD: endoscopic papillary balloon dilation, EPLBD: endoscopic papillary large balloon dilation.

**Table 3:** Factors associated with the occurrence of cholangitis in the IH group.

Factors	Cholangitis		
	+ (*n* = 8)	− (*n* = 14)	*p* Value
Age, median	71.5 (45–89)	66.5 (26–90)	0.29
Sex, male/female	5/3	11/3	
Largest stone size (mm), median	6.2	8.0	0.02
Number of stones			
1	1 (13%)	2 (14%)	0.007
2–5	1 (13%)	5 (36%)	
>6	6 (75%)	7 (50%)	
Untreated papilla	2	2	0.04
Papillary intervention			
EST	2	0	0.05
EPBD	3	2	
EPLBD	1	1	
None	2 (25%)	11 (79%)	
Prophylactic antibiotics	6	12	0.602

EST: endoscopic sphincterotomy, EPBD: endoscopic papillary balloon dilation, EPLBD: endoscopic papillary large balloon dilation.

## Data Availability

The datasets generated and/or analyzed during the current study are available from the corresponding author on reasonable request.
